# 5G-Enabled Autonomous Driving Demonstration with a V2X Scenario-in-the-Loop Approach

**DOI:** 10.3390/s20247344

**Published:** 2020-12-21

**Authors:** Zsolt Szalay, Dániel Ficzere, Viktor Tihanyi, Ferenc Magyar, Gábor Soós, Pál Varga

**Affiliations:** 1 Department of Automotive Technologies, Budapest University of Technology and Economics, 6, Stoczek utca, 1111 Budapest, Hungary; viktor.tihanyi@auto.bme.hu; 2 ZalaZONE Automotive Proving Ground, 1, ZalaZONE tér, 8900 Zalaegerszeg, Hungary; 3 Department of Telecommunications and Media Informatics, Budapest University of Technology and Economics, 2, Magyar Tudosok krt., 1117 Budapest, Hungary; ficzere@tmit.bme.hu (D.F.); soos@tmit.bme.hu (G.S.); 4 Ericcson Hungary, 11, Magyar Tudosok krt., 1117 Budapest, Hungary; ferenc.magyar@ericsson.com

**Keywords:** Digital Twin, 5G, real-life measurements, autonomous driving, V2X, SciL

## Abstract

Autonomous vehicles are at the forefront of interest due to the expectations of changing transportation for the better. In order to make better decisions on the road, vehicles use information from various sources: their own sensors, messages arriving from surrounding vehicles and objects, as well as from centralized entities—including their own Digital Twin. Certain decisions require the information to arrive with low latency and some of this information (such as video) requires broadband communication. Furthermore, the vehicles can populate an area, so they can represent mass communication endpoints that still need low latency and massive broadband. The mobility of the vehicles obviously requires the complete coverage of the roads with reliable wireless communication technologies fulfilling the previously mentioned needs. The fifth generation of cellular mobile technologies, 5G, addresses these requirements. The current paper presents real-life scenarios—on the M86 highway and the ZalaZONE proving ground in Hungary—for the demonstration of vehicular communication with 5G support, where the cars exchange sensor and control information with each other, their environment, and their Digital Twins. The demonstrations were carried out through the Scenario-in-the-Loop (SciL) methodology, where some of the actionable triggers were not physically present around the vehicles, but sensed or simulated around their Digital Twin. The measurements around the demonstrations aim to reveal the feasibility of the 5G Non-Standalone Architecture for certain communication scenarios, and they mainly aim to reveal the current latency and throughput limitations under real-life conditions.

## 1. Introduction

Autonomous and advanced technology in cars is currently changing the automotive industry. The effects of future transport technologies are intensively researched, and the innovation is blooming in the related domains. Increased road safety, streamlined travel time, customized facilities, increased energy management, and parking benefits deduce key social benefits.

An outstanding advantage of automation of automobiles is the improvement in safety, which comes naturally when we eliminate the root of most traffic incidents: human behavior on the road. Manufacturers have a wealth of experience with testing non-autonomous vehicles, and the procedure includes running millions of test kilometers that allow issues to be resolved and the specifications are clear, the vehicle has to be technologically ready for the road in compliance with all applicable regulations. The same procedure applies to autonomous vehicles, as they must comply with all sorts of laws and regulations that are usually the driver’s responsibility. A successful approach could be to follow the rule of the Netherlands, where the vehicle itself must have a license or certificate [1] similarly to human drivers’ license. It is mandatory to establish a standardized inspection procedure for the vehicles themselves to receive the license. This standard procedure includes a proving environment and a verification of test-conditions that can prove the vehicle’s overall (as well as specific) feasibility for traffic. Because of the various issues in testing on public roadway infrastructure with conventional traffic, this approach cannot be considered to be safe enough in all utility cases. Hence, it is necessary to build test sites and establish systems that allow autonomous vehicles to test with erratic traffic situations in realistic surroundings. Furthermore, a protected environment allows for the development of situations that are difficult to replicate or unsafe in real-life situations (e.g., near-accident affairs).

Meeting safety requirements and making decisions for multi-focal optimized driving is supported by sensors that are internal to the vehicle or that reside in the environment (including other vehicles). Furthermore, information is exchanged with the Digital Twin of the vehicle, since it can have additional information regarding traffic, roadwork, weather, and other conditions. The communication between the vehicle and all the other external entities are supported by wireless communication technologies, a prominent one of these is 5G, which offers end-to-end service guarantees.

This paper presents a state-of-the-art demonstration of the Scenario-in-the-Loop field-testing method for autonomous vehicle solutions, being supported by 5G mobile network services. The research team focused on the conceptual design and implementation of a demonstration that was internationally renowned in the field. The essence of the project is the cooperation with industrial partners; during the project, autonomous vehicles with 5G connection testing environment were developed with the support of the ZalaZONE [2] proving ground.

**Figure 1:**
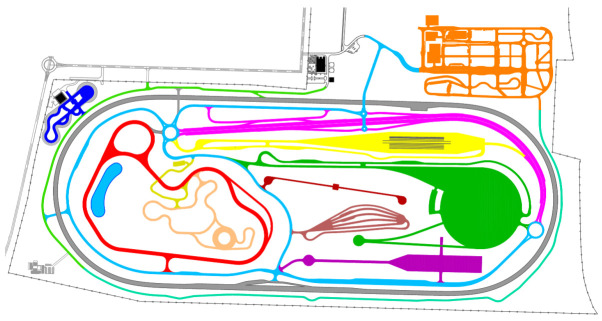
The 5G Scenario-in-the-Loop (SciL) driving tests were carried out in the smart city zone (top right section) of ZalaZONE proving ground.

Beyond being designed for connected and automated vehicle testing, ZalaZONE (see Figure 1) also incorporates all of the conventional vehicle testing elements for vehicle dynamics tests and endurance tests, such as the vehicle dynamic plate (marked with green color), or the high-speed oval (grey), or the handling course (red), or the braking platform (yellow). Furthermore, there are also unique elements, such as the smart motorway module (magenta) or the smart city zone (orange). The smart city zone is the one from the 8+1 unique testing propositions that ZalaZONE offers [3]. It is separated into five sub-areas. The low-speed parking zone (1) includes all of the surface parking facilities and it also incorporates a parking garage. The so-called high speed multi lane environment (2) is a 700-meter-long road segment with two times four lanes and several crossing roads. The downtown area (3) has relative higher building facades, which are made of real construction materials. The suburban area (4) has residential type building facades, with a lower height. This suburban area surrounds the downtown area and the high-speed multilane sections. Additionally, finally, there is the T-junction section (5) with 14 different T junctions that are challenging for the connected end automated vehicles.

This environment provides the physical infrastructure for performing Scenario-in-the-Loop (SciL) demonstration tests [4]. The concept of SciL tests can be aligned with the “Digital Twin” technology, which requires a highly reliable and low-latency cellular network infrastructure for autonomous vehicle control. Furthermore, autonomous vehicle use-cases have been defined in order to demonstrate the applicability of the 5G/4G LTE (Long Term Evolution) Advanced network connections.

The demonstrations presented in this paper took place in two areas, which are geographically close to each other. The 5G highway tests were carried out in the M86 highway at western Hungary, whereas the 5G-supported SciL demonstrations and measurements were taken place in the smart city zone of ZalaZONE (see Figure 1), which has been laser scanned previously, enabling the creation of the centimeter precision Digital Twin of the smart city zone in the simulated virtual space. The autonomous vehicle platform that was used in the scenarios travels autonomously, responding to situations that are based on its own sensors and information from the simulated virtual world [5].

The official opening of the ZalaZONE proving ground for connected and automated vehicles took place on 20 May 2019 in Zalaegerszeg, Hungary. Three test modules were opened for public use while the construction continues and the proving ground will be complete by the end of 2021. Since the summer of 2018, vehicle manufacturers and Tier 1 automotive suppliers have been organizing tests at ZalaZONE. The 8+1 unique testing propositions of the proving ground rely in their complexity, since they are not only suitable for performing conventional vehicle tests, but also support the special testing requirements of highly automated vehicles and autonomous driving functions. ZalaZONE also provides an excellent location to demonstrate, test, and validate new telecommunication solutions, especially connected vehicle functions [3]. Beyond the previously completed LTE advanced (4G+) coverage [6], a standard 5G test station was launched for the opening ceremony. It provided the low latency 5G communication network for the mixed reality testing demonstrations [7]. The M86 highway demonstrations and measurements were using the same 5G non-standalone architecture (5G NSA) as in the ZalaZONE area.

The novel contributions of the current paper are the following:

- It presents real-life 5G based V2X communication scenarios with actual network traffic measurement results—for which no public data are available yet.
- It presents novel Scenario-in-the-Loop measurements and results, where 5G communication link was necessary for providing a stable, real-time connection between the real world and its virtual representation. This means that the vehicle under test does exactly the same thing in reality and in parallel real-time in the virtual space. It responds real-time either to real obstacles on the test track or obstacles that are generated in the virtual space, such as the dummy during the demo.
- It presents latency, round-trip-time, and packet inter-arrival time results in these real-life scenarios under the 5G NSA architecture. Based on these, it provides a feasibility study on which the vehicular communication scenario can already be supported by this technology.

The paper is structured, as follows: Section 2 discusses the related work, presenting the expectations and real-life measurements in the topic of V2X and the demonstration methodology of the Scenario-in-the-Loop approach. In Section 3, the demonstration scenarios are discussed with the infrastructural background of the project. Section 4 describes the key elements of the SciL architecture. In Section 5, the 5G network architecture and measurement methodology are discussed. Section 6 presents the 5G measurement results, while Section 7 concludes the paper.

## 2. Related Work

### 2.1. Expectations and Feasibility of 5G in the Field of Vehicle Mobility

5G aims to be a key enabler of more reliable communication for vehicles, which will play a critical role in managing the safety challenges that come with vehicle automation and autonomy. The goal is to make the vehicles smarter and create a more secure driving experience [8]. This is achieved by covering the requirements for both enhanced Mobile Broadband (eMBB) and ultra-reliable Low Latency Communications (URLLC) applications [9].

5G will reduce the latency and increase reliability [10] when compared with current technologies, enabling new use-cases, such as trajectory sharing, real-time local updates, and coordinated driving. Vehicle-to-Everything (V2X) communication that is based on 5G will support latency at ten milliseconds end-to-end and one millisecond over the air—in the case of edge computing. Similarly, 5G provides very high reliability, targeting 99.999 percent for ultra-reliable transmissions [11].

The recommendation 3GPP TS 22.261 [12] sets generic requirements for 5G-based services, whereas 3GPP TS 22.104 [13] deals with the requirements of cyber-physical control applications in various vertical domains. This quasi-standard sets very harsh values for latency (ranging from 0.5 ms to 500 ms, depending on the use-case), high reliability (starting from “five-nines” to 99.999999%), and long-term reliability (with MTBF (Mean Time Between Failures) ranging from one day to 10 years). Besides these QoS (Quality of Service) metrics, KPIs (Key Performance Indicators) are also set for non-deterministic communication as well as for periodic and aperiodic deterministic communication. Other issues that are usually left for the telco vendors are also addressed this time—including mixed traffic scenario KPIs, clock synchronization, positioning performance, and issues of network operation.

While 5G is meant to satisfy machine-to-machine communication needs in general, V2X communication is the expected “killer application”. The QoS and KPI parameters related to 5G-V2X are detailed in 3GPP TS 22.186 [14]. Some of the metrics of use-case scenarios that are presented in 3GPP TS 22.289 [15] are also worth considering for V2X, although this recommendation specifically addresses rail communications red issues that fall outside the interest of the current paper.

The 5G architecture [16] was created with the earlier described requirements in focus.

In order to reach the required KPIs, the deployed infrastructure has to be fine-tuned and kept well-groomed—mostly autonomously. One of the necessary tasks here is setting up and keeping the service slices operational. This area is extensively researched and the competing ideas are continuously verified through simulations as the examples below.

Because low latency and high reliability are both key factors in V2X communication, their joint function should be investigated. The simulation results shown in [9] indicate that a maximum reliability and latency joint function value is dependent on vehicle density in 5G autonomous vehicular networks. To improve both the reliability and latency performance and implement URLLC, network slicing solutions need to be applied. One such method for 5G V2X is proposed by [9] that extends from resource slicing to service and function slicing.

As a current, practical solution for addressing high reliability, low latency, and even enhanced mobile broadband, ref. [17] proposes a programmable and dynamic end-to-end slicing mechanism in an M-CORD (Mobile-Central Office Re-architected as Datacenter) based LTE network. In the meantime, M-CORD has become integrated into COMAC (Converged Multi-Access and Core) [18]. It is noticeable that this solution uses the Non-Standalone (NSA) 5G architecture, with the currently available Evolved Packet Core (EPC), instead of the future 5G core. The solution utilizes one of the key features of M-CORD/COMAC, namely, the virtualized EPC enables customization and modification.

Because V2X communication has complex servicing needs, ref. [19] proposed a new paradigm of a 5G-enabled vehicular network, integrating the concepts of EC-RAN (Enhanced Cloud-RAN), D2D (Device-to-Device) communications, and SDN (Software Defined Networking) technologies. Their method aims to provide efficient and elastic services for mobile applications, which requires large bandwidth resource and high computing capability. The solution uses the cloudlet resource management approach, which also includes resource allocation and sharing.

On the other hand, non-centralized architectures for on-the-fly V2X provisioning also have advantages, as [20] demonstrated through a Road Side Unit (RSU)-based edge solution. Here, every RSU plays the role of a broker that performs job assignments. The proposed framework shows considerable improvements in terms of latency measurements and bandwidth utilization when compared with two competing solutions.

### 2.2. Real-Life Measurements Related to 5G-Supported V2X

The standards and the quality of service that are offered by mobile network operators need to be reinforced by accurate and relevant measurements for autonomous vehicles. The main question is whether the data transmission technology—5G—meets the needs of the autonomous car use-cases. In addition to the higher available bandwidth that is offered by 5G, another huge advantage is the seamless handover between cells without packet loss [21]. The bandwidth provided by 5G, both for the up- and downlink, offers significantly more options regarding use-cases. Sensor data can be transferred between an edge server and the vehicle real-time; hence, the V2X approach of efficient collaboration between environment monitoring and mobile edge computing becomes reality.

In general, the environment of the 5G-targeted vehicles can be very diverse, ranging from slow-moving industrial AGVs (Automated Guided Vehicle), through urban traffic scenarios with great number of endpoints, to vehicles moving on a high-speed motorway. Potentially, 5G-supported V2X use-cases (including not only cars but drones) are gathered well in [22]. While V2X and 5G standardization are still ongoing, early adaptations of the technology set are already available and deployed (i.e., 5G NSA). While real-life, application-related 5G measurement results start to appear, different fields, such as IoT [23] or healthcare [24], comprehensive reports of the current state-of-the-art “on the road” (i.e., 5G-capable automated vehicles on motorways) are still missing. The authors of [25] describe a 5G V2X testbed already as early as in 2016, when 5G standardization was in the requirement study phase at 3GPP; and, indeed, the paper merely discusses radio-subsystem details and capabilities.

The performance of adaptive beamforming was analyzed in relation to 5G V2X communication in [26], although this study was based on simulations, and merely considered the radio-link performance. Note that our real-life measurement scenario also used beamforming as the feature of the used actual equipment—and our measurement also involved the core network—not to mention the physically moving vehicles on a motorway. While various 5G V2X application scenario-based papers are available, they mostly focus on optimizing communication (such as collision avoidance [27] or platooning [28], rather than on network traffic capabilities—not to mention real-life measurement results—which provides the uniqueness of the current paper.

The protocol sets for automated vehicles are still changing [29]. The various use-cases would also generate diversified data needs, where different throughput, latency, jitter, packet loss, and other SLA parameters will be critical. From a mobile network operator point of view, network slicing will be the solution to serve these needs [30,31]. Network slicing can help to prioritize traffic during end-to-end data transmission. A time-critical data stream will get higher priority over a static, but non-interactive, stream. Furthermore, the critical data stream can even receive latency guarantees [32].

### 2.3. The Concept of Scenario-in-the-Loop—SciL

Autonomous vehicles (AVs) are essential elements of the potential transportation, and this is no longer just a vision: there are companies that have the license to test their driverless cars in real traffic without any human involvement in the vehicle [33]. The various testing approaches for V2X are surveyed by [34]. However, these kinds of experiments focus primarily on a single vehicle’s characteristics without using the advanced AVs facility, when considering the importance of the system and other vehicle cooperation. At present, the so-called “in-the-loop” approach appears to be the most powerful technique for measuring the interaction between AVs and the control system. The various virtual environment approaches for testing are becoming commonly acceptable and used in practice. These include Hardware-in-the-Loop (HiL), Software-in-the-Loop (SiL), Vehicle-in-the-Loop (ViL), or Scenario-in-the-Loop (SciL) [35]. Regarding the Scenario-in-the-Loop (SciL) testing environment [36], not only the vehicle’s physical attributes are evaluated, but its sensors are also tested by virtual twin realization. In this case, the scenario being studied is simulated and partly realized in parallel.

**Figure 2:**
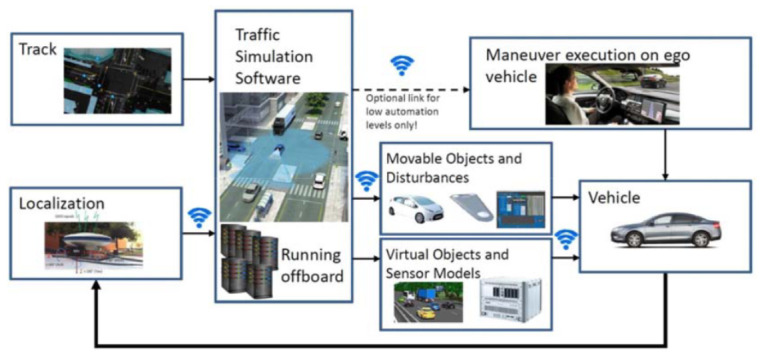
The Scenario-in-the-Loop (SciL) testing concept [4].

The SciL concept is visualized in Figure 2. The physical vehicle moves on a physical track—be it either a test-track, proving ground or actual traffic—and responds physically on both simulated and real environmental information. This also means that some or all of the environmental “obstacles” and “events” actually happen only around the Digital Twin (simulated), but the real vehicle also responds through its movements.

A definite advantage of this newly proposed solution is that the SciL methodology connects physical and virtual testing real-time, with high fidelity, through exploiting the capabilities of the latest generation mobile communication technology. This allows for the testing of autonomous vehicles to be more creative, thorough, and repeatable. The usage of varied moving obstacles and complex scenarios at the Digital Twin are to be acted upon in the physical setting by the autonomous vehicle—and this environment can be repeated over-and-over again in an exact way, because the scenario is recorded in the Digital Twin.

Further emerging challenges—that include sensor models and vehicle models—must also be resolved when designing autonomous vehicles [37]. Additionally, vehicle technologies that are focused on simulation inevitably require X-in-the-Loop validation and verification processes [38]. This proposes that the implementation of a SciL framework is not only advantageous, but necessary. The missing test-experience of self-driving vehicles can be fulfilled in a closed environment of smart cities. With the aid of SciL, various scenarios can be developed that otherwise could only be tested in real traffic [39].

## 3. Demonstration Scenarios

The demonstrations that are presented here have two parts.

1 In the first part of the test, the solutions of another Hungarian automotive organization were presented, partly using the technologies and tools that were used in the previous presentation, but combined and further developing them. The exact position data were supplemented with vehicle environment sensors. Furthermore, information from the Digital Twin also influenced vehicle behavior—in here, the information exchange that is involved the 5G NSA architecture, for which we also measured the network performance.
2 In the second part, the vehicles were moving in a temporarily closed segment of the M86 highway of Hungary. During this setup, the data were gathered through various roadside and vehicle-internal sensors, while 5G NSA measurements were carried out for analysis.

### 3.1. BME Self-Driving Simulation

**Figure 3:**
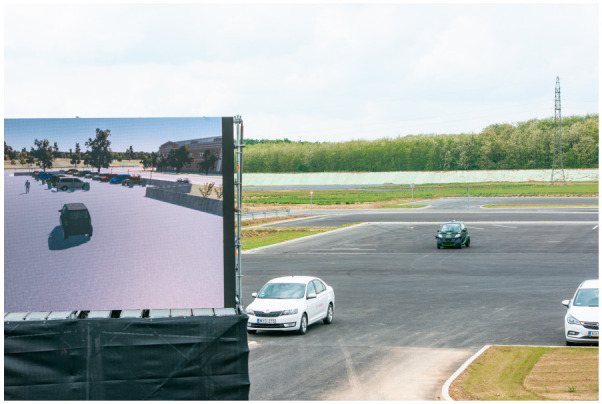
The VUT and the virtual environment.

In this case, the demo was carried out with one autonomous test vehicle (VUT, Vehicle Under Test); however, pedestrians and other vehicles also appeared as traffic obstacles. During the autonomous operation, the car travels in the same way in the virtual space as in the simulation, but this environment may differ from the one on the real test track (see Figure 3).

**Figure 4:**
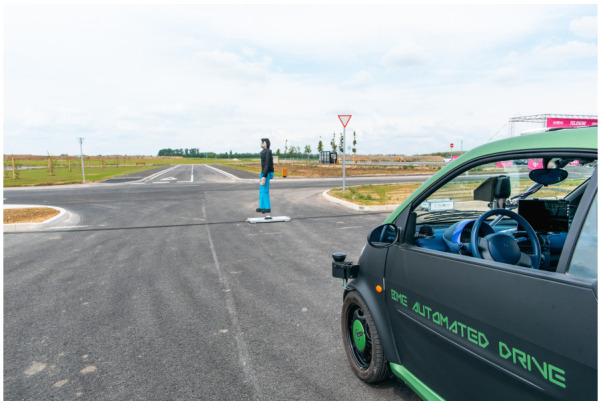
The VUT automatically stops while the 5G controlled dummy “walk” accross the road.

In the virtual space, objects (pedestrians, vehicles) have the same effect on the operation of the vehicle as those detected by its own sensors. They are injected into the vehicle control system besides the signals of the physical sensors. In addition to virtually created objects, real objects—dummies and vehicles—can also be controlled by the SW system and be detected by the car’s sensors. The communication between the vehicle, the simulation environment, and the controlled test objects was realized via a 5G network ensuring near real-time connection. Two scenarios were presented with the test vehicle. The first was a feature called “Valet Parking”: the vehicle automatically parks for a phone call and goes to the caller. Meanwhile, during the demo, a virtual and then a controlled pedestrian crossed the path of the vehicle (see Figure 4).

As expected, the VUT stopped in front of them and continued its journey only after the obstacle had disappeared. The second scenario is a Traffic Jam Pilot. Here, the car follows the lanes that were detected by its cameras, steering in self-driving mode and adjusting its speed to the vehicle in front of it. During the demonstration, the VUT first followed a virtual vehicle and then a real car. In both scenarios, the test vehicle operated in a fully self-driving mode.

The detailed test scenario is as follows:

1 enter parking space;
2 leaving parking space;
3 stop in front of the virtual pedestrian while turning;
4 stops in front of the real pedestrian;
5 after turning, VUT stops behind the virtual vehicle, then overtake it; and,
6 turn back, follow the real vehicle, then stop.

### 3.2. Measurement Campaign on M86

The second demo—and the corresponding 5G measurement—was a part of a measurement campaign that was close to the ZalaZONE track. This campaign was carried out on a highway section of M86, nearby the town of Csorna, in the North-Western part of Hungary (Győr-Moson-Sopron county, Western Transdanubia Region). The goal of the measurement campaign was to record vehicle and infrastructure data being essential to the implementation of the technologies to be developed in later phases of the R&D projects in the area of automated vehicles. This campaign helped to test features and collect data in real conditions instead of the laboratory environments. Several universities and automotive companies of four countries participated in the measurement campaign.

**Figure 5:**
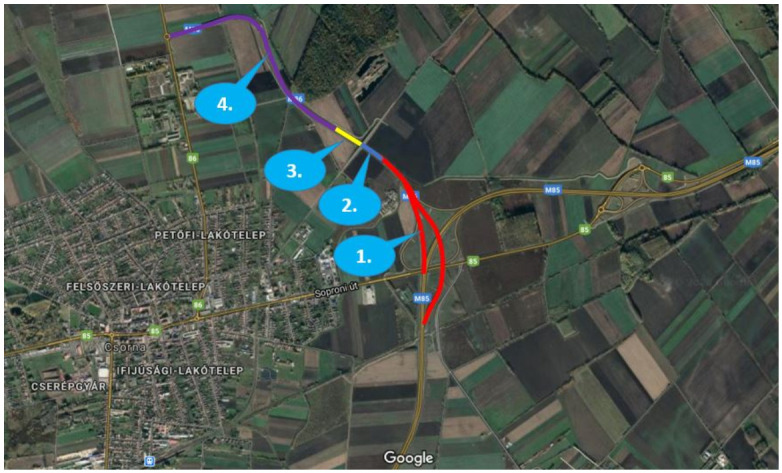
Sections of the test site created by closing a 3.5 km long segment of M86 Motorway from public traffic.

The road section that is shown on Figure 5 was completely closed from traffic, only the measurement vehicles were allowed to use the road. All of the vehicles had a GNSS (with 1 cm accuracy) transmitter so that the dynamic parts of the scenarios were fully detected automatically. An essential goal of the campaign was to realize measurements fully comparable, i.e., data were detected by vehicles and by sensors located in the infrastructure. Meanwhile, the communication architecture (5G cellular network) was continuously tested on vehicles and infrastructure both in terms of latency and data transmission with ex-post evaluation.

The measurements were using mainly sections 1 and 2 of both carriageways at the same time. The 5G-related measurements were run in section 1 only. There was also 4G coverage on the testing area; therefore, on sections 2, 3, and 4, the UE (User Equipment) fell back to 4G. Besides the physical infrastructure, a high bandwidth fiber optic communication network was available along the entire section, and several sensors, cameras, and Variable Message Sign (VMS) gantries were deployed with a fixed power supply. Using this infrastructure, additional sensors (cameras, laser scanners, etc.) and mobile 5G base stations were deployed during the tests.

## 4. SciL Demonstration Architecture

### 4.1. High-Level Architecture of the SciL Demo System

**Figure 6:**
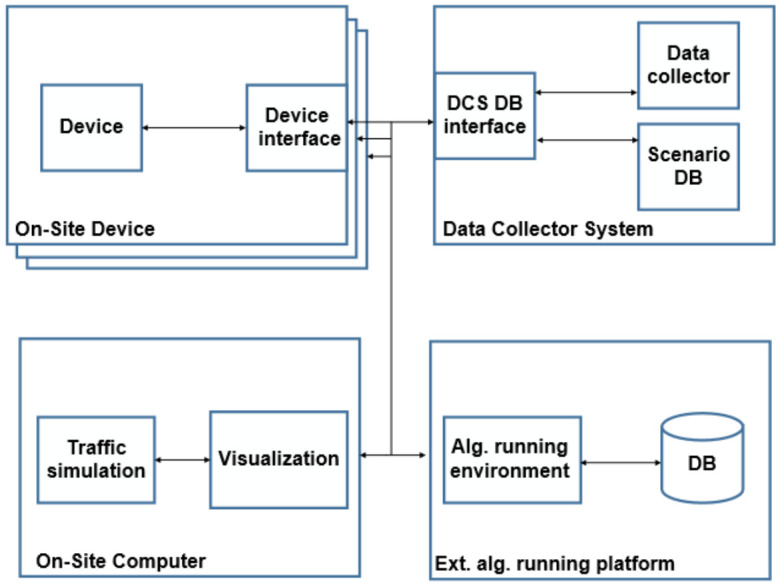
High-level architecture of the communicating systems and their modules.

The Scenario-in-the-Loop demonstration system is built in a modular manner, and it includes on-site elements as well as central modules. The logical elements and their connections are depicted in Figure 6 as a high-level architecture. The logical elements are the following:

- devices: data provider or consumer on-site devices—during the demonstration, these included the Vehicle Under Test, road-side-unit sensors, and a physical pedestrian dummy;
- on-site computer: the computer operating the SciL simulation and realizing the Digital Twin; it also receives data directly from the devices and the Data Collector System (DCS);
- data Collector System, DCS: the main task of the DCS is to collect and distribute various data in the system; and,
- external Algorithm Running Platform: There could be external elements receiving and analyzing the data made available through DCS to provide further insights (e.g., for vehicle manufacturers, testers). The current demonstrations have not provided data externally.

Regarding devices, it is difficult to visualize the structure of internal blocks due to their diversity. In order to provide high flexibility for SciL verifications, all of the devices must be able to connect to each other or to the DCS via a predefined interface. Each device has a task manager that manages its internal processes, which is different depending on the device type. The test vehicles themselves—that are used in the demo—are suitable for autonomous driving up to SAE level 4 automation. Moreover, the vehicle simulating traffic operated in fully automatic mode (SAE level 5), according to the predefined traffic scenario.

### 4.2. The On-Site Computer

**Figure 7:**
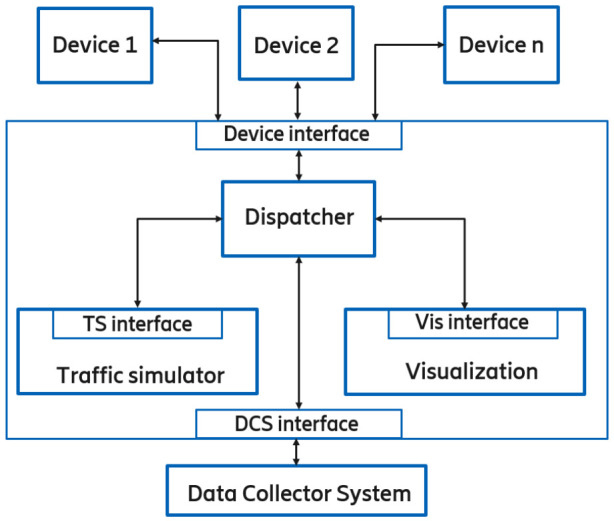
On-site computer architecture.

The task of the on-site computer is to run computationally intensive tasks and realize the Digital Twin. Its internal modules and external interfaces are depicted by Figure 7.

The Traffic simulator sub-module generates virtual traffic around the VUT and displays it in the simulation environment in order to test the responses of the virtual vehicle.

In the Visualization subsystem, the virtual reality of the traffic simulator and the detected environment are displayed. The purpose of this subsystem is to present the scenario realistically.

The Dispatcher subsystem transfers the received data to the desired subsystem through handling the processing, conversion, and transmission of messages from additional components. While, the on-site computer communicates with the devices participating in the scenario (VUT, dummy, road-side-unit sensors, etc.) via a device interface. It sends the appropriate control commands, position of virtual objects, and receives the signals and objects that are detected by each device, as an input of the Traffic simulator or the Visualisation subsystem.

The on-site computer receives the desired (traffic) scenario from the DCS interface and forwards the signals generated in the virtual space to the VUT—hence creating the Scenario-in-the-Loop.

### 4.3. SciL System Requirements

The SciL system has a number of requirements as a pre-condition for successful implementation. In the case of the demonstrated system, we can distinguish between high priority and less high priority data. High-priority data must be delivered to the destination in near real-time or with very low latency. Regarding hard-real-time scenarios, high latency or packet loss could have catastrophic effects, such as failure to break, thus leading to safety issues. On the other hand, in the case of softer requirements, failure to deliver the message will not have serious consequences, but the quality of service will deteriorate (e.g., ending up with degraded video stream quality).

Table 1 summarizes the main differences on requirements regarding hard-real time and soft-real-time message transfers for the SciL system.

The requirements for data transmission between the elements are as follows:

- important data must be able to be transferred from devices to on-Site Computers in near real-time;
- important data must be able to be transmitted to the Data Collector System (DCS) in near real-time;
- less important data must be transferred from devices to On-Site Computers; and,
- less important data must be transferred from the devices to the DCS.

Further functional requirements for the overall system includes the following:

- the received data is stored in the database by the DCS;
- the received data have to be transmitted to the target devices via a dispatcher service;
- data service must be provided for the External Algorithm Running Platform; and,
- log events have to be recorded.

### 4.4. Availability

Availability is defined as the ability of an application or resource to function as intended, presented in percentage. In the case of a SciL system, as many data can be catastrophically affected by the latency or packet loss of the system, very high availability must be assured, and the related systems must detect the failure of service and be able to handle the situation. A typical example is that the scenario should not start if the system detects an error in the data communication, rather than cause an accident with a possible failure to brake. In the case of the SciL architecture, in addition to availability, operational safety is also a crucial indicator of the system. In the present case, it is not critical that the system does not work, but it must be reliable when it does. The system’s operational security means that the system processes and responses to requests in a given time are defined by the specification. During the design and implementation of such a system, we keep in mind the following approaches:

- Avoid Error—apply development methods that exclude or at least minimize errors.
- Troubleshooting—applying testing procedures that significantly increase the chances of errors being detected before you start using the system.
- Fault Tolerance—the use of design methods that ensure that errors can be detected and managed.

## 5. 5G Network Measurement Scenario

### 5.1. 5G Network Configuration and Architecture

In the demonstrated system, various technologies naturally separate wireless communication (5G-based V2X) from the in-vehicle communication system (CAN bus). Information from/to in-vehicle modules that appear on the CAN bus need to get filtered and passed to/from the internal 5G modem. This requires a dedicated device in the vehicle, which is capable of processing and transmitting CAN traffic. There were positioning, camera, and ECU (Engine Control Unit) data, as well as other vehicles’ data shared between vehicles and with their Digital Twins via the 5G network.

Table 2 summarizes the types of data that were collected from the various sensor devices.

Some of the devices used during the demonstrations were not yet commercially available at the time (and some still continue to be that way). These were “precommercial” devices and, since the 5G frequency tender was not prepared in Hungary at the time of the demonstration, the used 3.5 GHz frequency range was available merely in test-operation.

In the test track area, Ericsson’s 5G antenna system was installed on Magyar Telekom’s telecommunications tower for the demo, together with Ericsson’s mobile-core network solution, which was connected to their headquarters in Aachen. This provided the experimental 5G network. The on-site computer was used as part of the Mobile Edge Computing (MEC) infrastructure in order to control and manage the demo scenarios. The fact that these devices situated near the network cell controllers (eNB, gNB) enabled to provide the required computing capacity together with extremely low latency. The vehicles under test were equipped with 5G NSA modems (WNC 5G Pocket Router) in order to provide connectivity between the control center and in-car localization over VPN. T-Systems provided broadband optical connection, as well as the physical infrastructure. Additionally, Magyar Telekom’s full 4G+ coverage was continuously available as a back-up solution as part of the normal commercial network. The 5G network was made available by engineers from Ericsson, Magyar Telekom, and T-Systems Hungary.

### 5.2. Physical Architecture of the Devices

**Figure 8:**
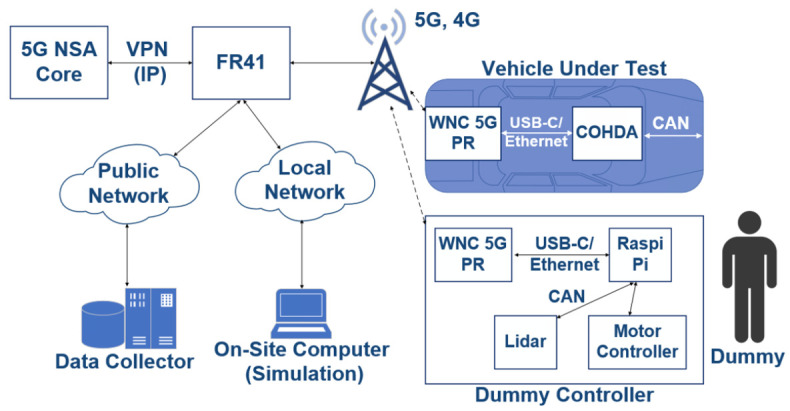
Communication architecture of the system.

The simultaneous operation of the 5G and 4G networks on the ZalaZONE proving ground was made possible by Ericsson’s Flight Rack device "FR41". There was a WNC 5G Pocket Router in every vehicle, which were capable of connecting to the FR41, as shown by Figure 8. The router has a 5G uplink channel and it also creates a local WiFi network so the in-vehicle equipment has IP-connectivity. Furthermore, the Cohda and Raspberry Pi devices were connected to this router via a USB-C Ethernet converter.

The main technical units of the demonstration were the following:

- Smart Fortwo passenger car: the car has undergone individual modifications to provide full-by-wire control—BME development—steering, gas, brake, etc., they are controlled by a special computer placed in the car;
- SciL virtual simulation environment, development of BME with the participation of ZalaZONE Research Team;
- In-vehicle support devices for autonomous mode:
  - iMAR positioning system;
  - one piece of a camera;
  - two pieces of LIDAR;
  - one piece of radar equipment.
- one electronically controlled movable Pedestrian Dummy, a development of the ZalaZONE Research Team together with BME;
- one ordinary car to display the traffic situation;
- Ericsson 5G modems; and,
- The 5G test network.

The test vehicles demonstrated real-time control situations based on predefined scenarios generated with OpenScenario [40].

The vehicles are commercially available mid-range passenger cars, converted to Traffic Simulation Vehicles (TSV) and Vehicles Under Test (VUT). The TSV has the self-driving capability, and both vehicles are equipped with the iMAR iTraceRT-MVT-510 type INS/GNSS reference system. Accordingly, the converted TSV can travel both in regular road traffic, with conventional driving, and in suitable areas in self-driving mode. Thus, the self-driving function of the vehicles can be switched off at any time. The devices interfering with the control of the vehicles are connected to a CAN bus, so they can control the electronically controlled steering gear, brake, and throttle. By disconnecting the built-in control unit from the CAN bus, the vehicle can be driven in the traditional way, as there are no “hard” conversions in the vehicle’s controls, it can travel as an ordinary vehicle anywhere in traffic. This allows for test vehicles to arrive at the test sites “on their own two feet”, where they can reconnect self-driving support systems.

### 5.3. 5G Connectivity Architecture

**Figure 9:**
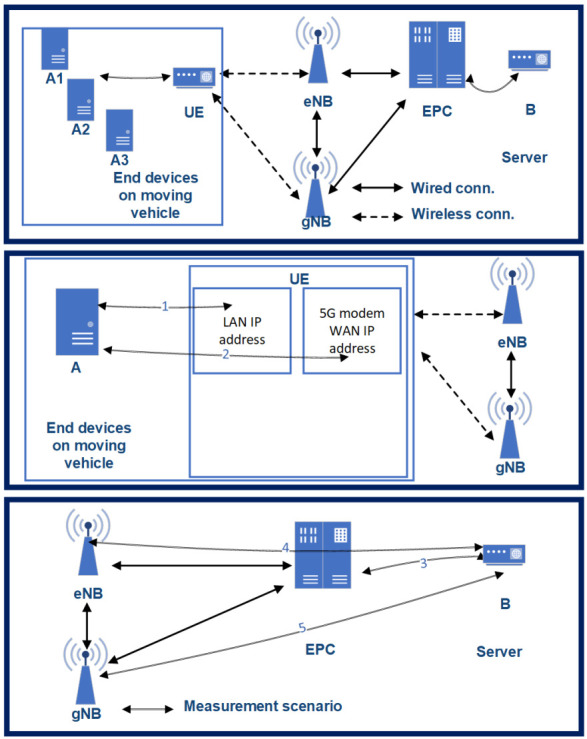
The architecture of our 5G testbed on the vehicle—as Option 3X.

The real-life 5G V2X measurements were executed in the architectural setup of Figure 9. A non-commercial 5G modem—still under development phase—was used for the measurement, while the packets were generated on three end devices. One of them was a dedicated traffic generator with microsecond accuracy; the other two were Raspberry 4s. The measurement traffic was generated on these three devices (A1, A2, A3), which were placed in a moving vehicle. The used network architecture was the Option 3.X, according to the standard described in the 3GPP NSA 5G network [41]. Packets starting from A reached the UE modem on a gigabit Ethernet connection, which was responsible for connecting to the 5G network. Data transmission packets from the UE to the server B direction always traveled through the eNB. From there, they moved on to the EPC to reach server B. Between the eNB/gNB and the EPC there was a long-distance, not only optical connection. From the server B backward, the packets traveled on a wired connection through the EPC to the gNB. Finally, the UE returned packets to the end-devices via Gigabit Ethernet.

As we mentioned, the examined mobile network architecture was a 3GPP 3.X 5G solution. Therefore, only the downlink channel used 5G connection, so the end-to-end RTT (Round Trip Time) is not a representative value for describing the 5G capability of the architecture. Furthermore, one-way downlink latency does not describe the characteristics of the network properly, just partially. The most crucial parameter to control is the latency between the UE and the gNB (practically, the radio link). However, we cannot measure this connection directly either, as the protocol encapsulation does not make it possible. Therefore, we measured the one-way latency, RTT—the sum of uplink and downlink latencies—and some other supplementary connections to calculate the latency between the gNB and EPC. The supplementary connections—according to Figure 9—are as follows:

- one-way latency ($T_{0}$): latency between (*l-bw*) device A1 and server B.
- Connection 1 ($T_{1}$): latency between the UE’s LAN interface (i/f) and the end-devices.
- Connection 2 ($T_{2}$): latency between UE’s WAN i/f and end-devices.
- Connection 3 ($T_{3}$): latency between EPC SGi i/f and server B.
- Connection 4 ($T_{4}$): latency between eNB’s s S1U i/f and server B.
- Connection 5 ($T_{5}$): latency between gNB’s S1U i/f and server B.

### 5.4. Measurement Methodology

The measurements described here were performed under varying radio conditions. We chose a state space approach to characterize some key parameters of the 5G network. We have measured the latency, while the car speed, the Inter-Arrival-Time (IAT) between the packets, and the Packet Length (PL) varied. These metrics are amongst the most fundamental attributes in the future 5G vehicle use-cases, as most of the V2X use-cases have strict time-critical requirements.

Based on our previous works shown in [23], we identified three measurement scenarios with different PL and IAT parameters. For comparisons, one reference scenario was also measured with constant parameters. The examined state-space scenarios are as follows:

- Scenario 1: from 2 ms IAT and 60 Byte PL to 62 ms IAT and 960 Byte PL, increment the PL by 60 Byte PL for every iteration and the IAT by 20 ms for every fourth iteration;
- Scenario 2: from 10 ms IAT and 250 Byte PL, to 310 ms IAT and 4000 Byte PL, increment the PL by 250 Byte for every iteration and the IAT by 100 ms for every fourth iteration;
- Scenario 3: from 10 ms IAT and 700 Byte PL, to 610 ms IAT and 11200 Byte PL, increment the PL by 700 Byte for every iteration and the IAT by 200 ms for every fourth iteration; and,
- Const: 2 ms IAT and 40 Byte PL.

Based on preliminary measurements [23], we found that the maximum MTU size on the network is 1450 Byte without segmentation. Still, as measurement scenario 2 and 3 suggests, we also used packets with PLs that were greater than 1450 Byte to characterize this kind of network behavior.

### 5.5. Graphical Presentation

Regarding the graphical representation of the results, we use the *box and whisker* plot. We chose this, because it shows, very well, the distribution characteristics of our data. Practically, it shows the five-number summary of a set of data: including the minimum score, first (lower) quartile (Q1), median, third (upper) quartile (Q3), and maximum score (excluding outliers). Moreover, box plots are useful in providing a visual summary of the data enabling researchers to quickly identify mean values, the dispersion of the data set, and signs of skewness.

## 6. Measurement Results

The main parameters of the described 5G Option 3.X scenario have been defined through a series of measurements. As descibed, this is a Non-Standalone 5G architecture, which uses the LTE EPC, and LTE eNB for uplink radio communications. In order to present a reference comparison, we have carried out similar measurements on earlier 4G [23]—and those results also apply here, since we used the exact same 5G environment. The aggregated results (Table 3) show that, for peak rates, the 5G scenario performs much better than 4G. As presented, 5G-NSA uplink transmission is an exception: it has similar performance as 4G, because, in here, the same eNB is used as for the 4G cases (but signalling is different). The comparative latency results are also significantly better for 5G than 4G, as expected.

### 6.1. End-to-End RTT Results Based on the 5G NSA Option 3.X Architecture

**Figure 10:**
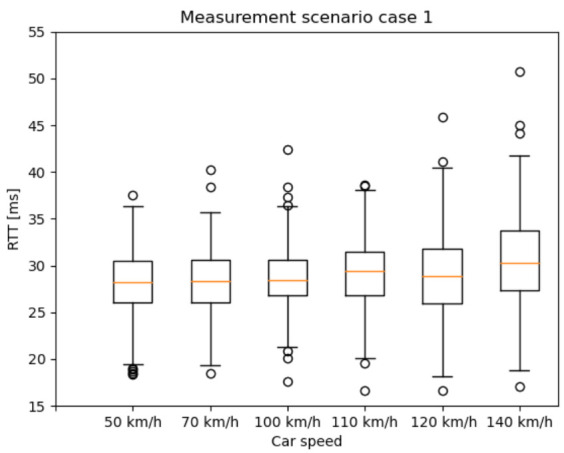
Round Trip Time (RTT) results with different car speeds—scenario 1: from 2 ms IAT and 60 Byte PL to 62 ms IAT and 960 Byte PL, increment the PL by 60 Byte PL for every iteration and the IAT by 20 ms for every fourth iteration.

**Figure 11:**
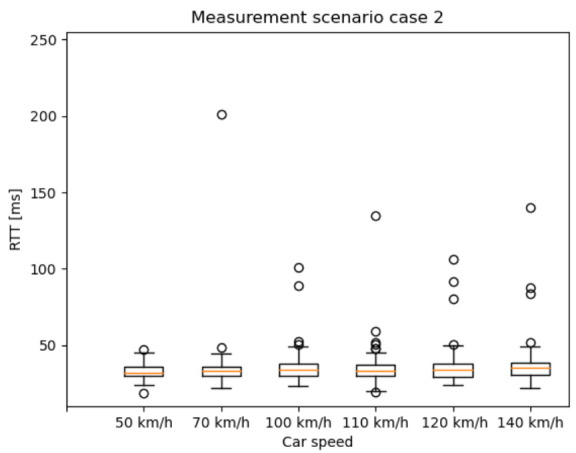
RTT results with different car speeds—scenario 2: from 10 ms IAT and 250 Byte PL, to 310 ms IAT and 4000 Byte PL, increment the PL by 250 Byte for every iteration and the IAT by 100 ms for every fourth iteration.

**Figure 12:**
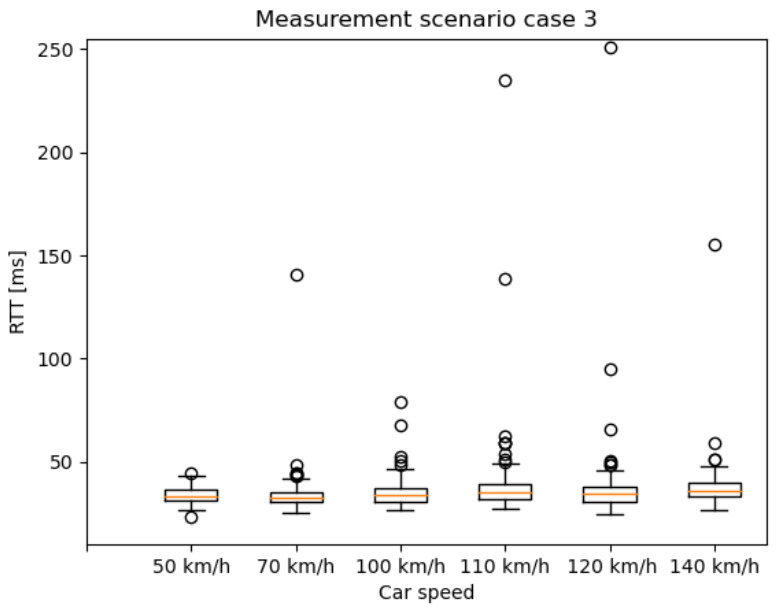
RTT results of measurement with different car speeds—scenario 3: from 10 ms IAT and 700 Byte PL, to 610 ms IAT and 11,200 Byte PL, increment the PL by 700 Byte for every iteration and the IAT by 200 ms for every fourth iteration.

In this section, the RTT results are presented as box plots in the different measurement scenarios. As expected—especially in the case of scenario 1 and 2 (Figure 10 and Figure 11)—the median values are increasing as the car speed rises. Additionally, for scenario 2 (Figure 11) and scenario 3 (Figure 12), the outlier values are more spread, because the packet length was much higher than in scenario 1.

**Figure 13:**
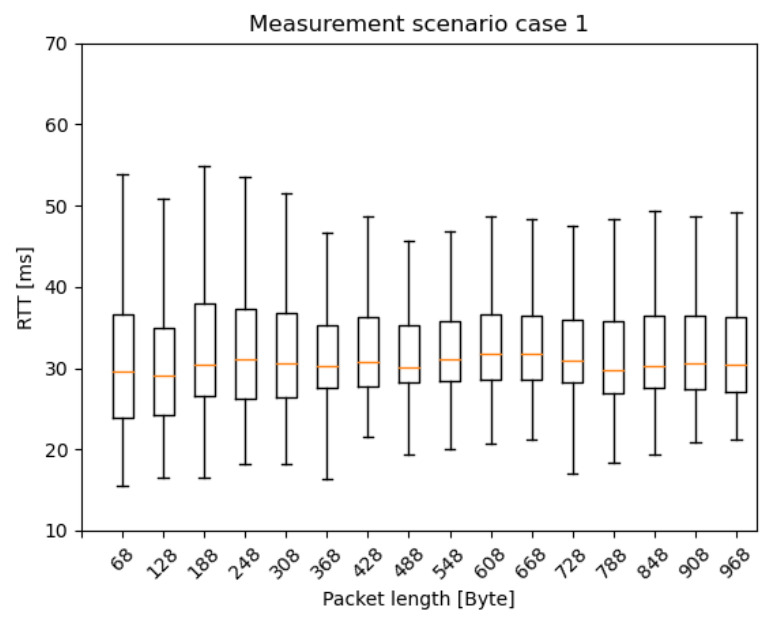
RTT results with different packet lengths—scenario 1: from 2 ms IAT and 60 Byte PL to 62 ms IAT and 960 Byte PL, increment the PL by 60 Byte PL for every iteration and the IAT by 20 ms for every fourth iteration.

**Figure 14:**
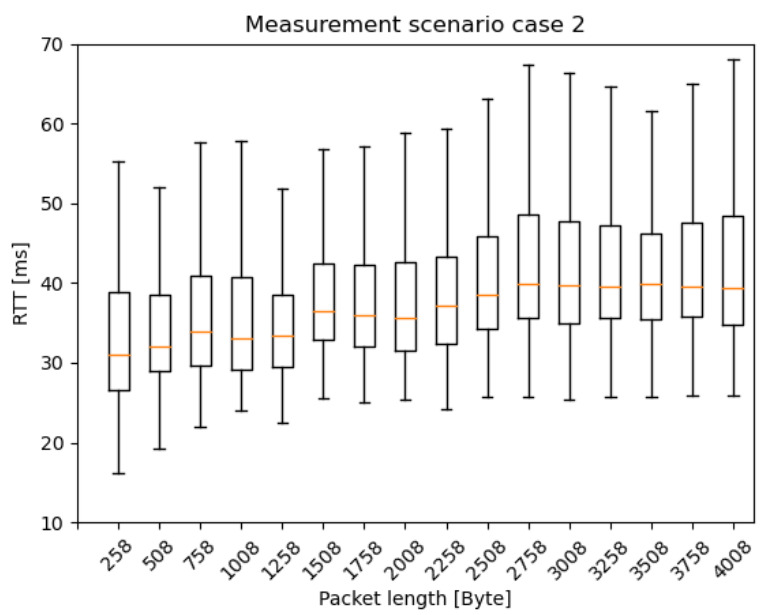
RTT results with different packet lengths scenario 2: from 10 ms IAT and 250 Byte PL, to 310 ms IAT and 4000 Byte PL, increment the PL by 250 Byte for every iteration and the IAT by 100 ms for every fourth iteration.

**Figure 15:**
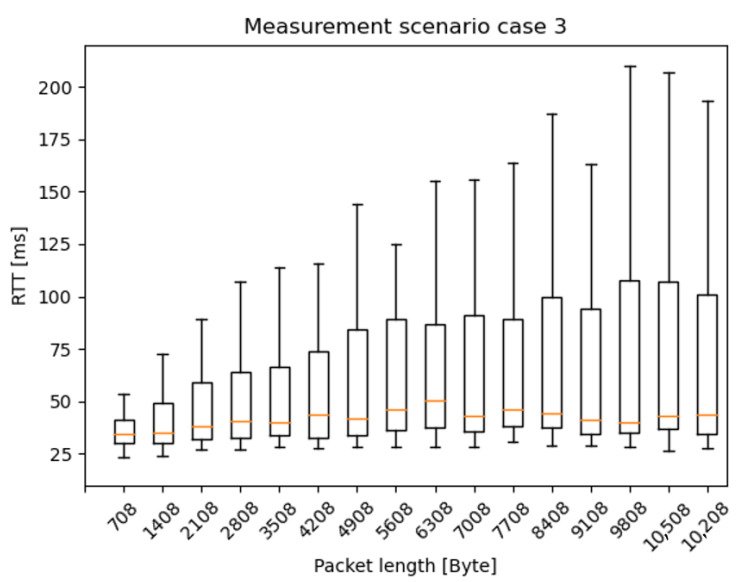
RTT results with different packet lengths—scenario 3: from 10 ms IAT and 700 Byte PL, to 610 ms IAT and 11,200 Byte PL, increment the PL by 700 Byte for every iteration and the IAT by 200 ms for every fourth iteration.

Figure 13, Figure 14 and Figure 15 present the RTT distribution in the case of the different packet lengths. Figure 13 shows no such trend as the previous one; however, in Figure 14 and Figure 15, an apparent skewness can be seen in the latency distribution as the Q3, and the maximum scores increase while the packet length rises. It is not a surprise that there is a positive skewness at large packet lengths as these packets become segmented. The median and Q1 and minimum scores stay identical in every scenario, as there are physical limitations to the lower limit of latency, such as the delay of the active device CPUs, NIC buffers, adapt packet to copper, optical and optical links, and so on. Regarding IAT results, there was no clear trend in the latency distribution, so it is not presented in this paper.

### 6.2. Calculating Average Radio Link Latency in the Real-Life Scenarios

The e2e RTT measurement results show high values when compared to similar 5G and experimental networks. The primary reason is the 3GPP 3.X NSA 5G architecture: only the downlink used 5G New Radio. Furthermore, we examined the latency between the network elements of the presented architecture (Figure 9). We received some assistance from the mobile core and radio network side to measure the latency on connections 3, 4, and 5—and learned that the network elements are physically far apart, and some transport elements are not presented in the architecture in Figure 9.

Although these network elements do not play a significant role in the 3GPP architecture, they add latency in the data transmission paths:

- Connection 1 ($T_{1}$): the A1–A2–A3 end-devices were connected to the UE via a Gigabit Ethernet switch. The latency between the A1–A2–A3 devices and the switch was less than 0.2 ms, so, for the measurement, the A1–A2–A3 devices can be considered as an A device.
- Connection 2 ($T_{2}$): surprising values were measured in the transmission between the A end-devices and the UE 5G WAN interface. In this connection, packets generated by the end-devices must pass through the UE via Network-Address-Translate or Packet-Address-Translate, which is performed by the UE, utilizing CPU resources. Although the UE’s CPU load was low (20%) throughout the tests, this link adds significant latency to the e2e latency. The value of this latency was 1.9 ms and the jitter was 0.8 ms.
- Connection 3 ($T_{3}$): the number of active devices in this connection is minimal. Each data path has at least 10 Gbps of optical data transfer capability. Based on the measurements at latency 2.1 ms, the jitter is less than 0.01 ms, regardless of packet size.
- Connection 4 and 5 ($T_{4}$, $T_{5}$): eNB and gNB perform a logically separate task in our measurement layout, but they are physically the same device with the same network connection. The eNB/gNB and the EPC, on the other hand, were physically quite far apart, about 140km, and were used for a variety of optical tracks, microwave sections, and even gigabit copper bridges with several different active L2-L3 devices. The data rate over the entire connection is at least 1 Gbps, latency 5.7 ms and jitter less than 0.9 ms, regardless of packet size.
- End-to-end RTT ($T_{RTT}$): the average RTT of the different measurement scenarios is 29.8 ms.
- End-to-end one-way latency ($T_{0}$): the average one-way latency—on 4G connection—from A1 to B is 17.8 ms.

After defining the needed supplementary measurements, where the latency *of the radio network*, the link between the gNB and UE, ($T_{gNB-UE}$) can be calculated:

(1) $$T_{gNB-UE}=T_{RTT}-T_{0}-T_{5}-T_{2};$$

where ($T_{0}$), is the latency between device A1 and server B; ($T_{5}$) is the latency between gNB’s S1U i/f and server B, and ($T_{2}$) is the latency between the UE’s WAN i/f.

Calculating with the *average* measurement values above, we get the following rough value:

(2) $$T_{gNB-UE}=29.8\;\mathrm{ms}-17.8\;\mathrm{ms}-5.7\;\mathrm{ms}-2.1\;\mathrm{ms}=4.2\;\mathrm{ms};$$

Nevertheless, the resulting ca. 4.2 ms latency between the gNB and UE is just an approximation, as the used latency values come from different sources and measurement methods. However, such a result is aligned with the expectations—we have not applied any traffic shaping, slicing, or URLLC fine-tuning methods—and most of the future use-case requirements of 5G V2X use-cases.

### 6.3. 5G E2E Reference RTT Measurement with Symmetric New Radio Resources

**Figure 16:**
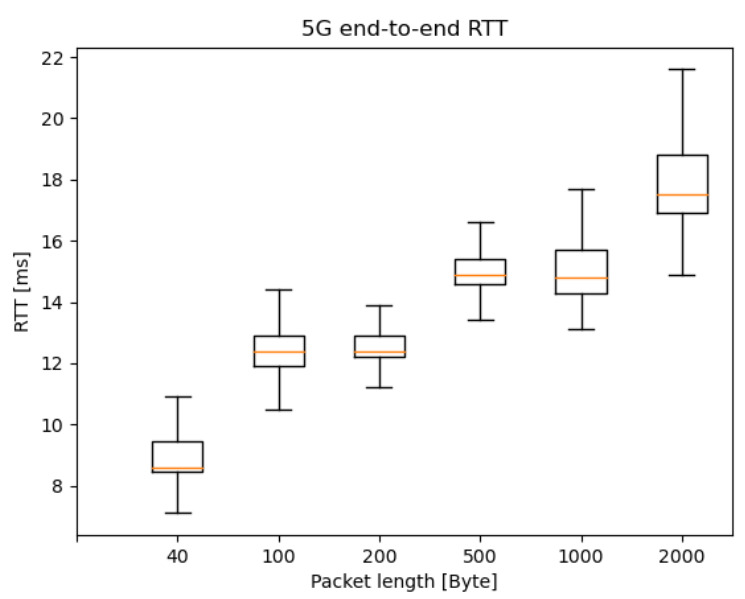
End-to-end 5G RTT results with different packet lengths—both the uplink and downlink uses 5G gNB; hence, one-way-delay is approximately half of the RTTs presented.

In order to eliminate some asymmetric effects (usage of eNodeB and gNB for uplink and downlink, respectively), we have performed independent reference measurements on the network of a large MNO’s 5G network in Hungary. In contrast to the M86 NSA 5G network, this architecture provided 5G connection on both the uplink and downlink channel, and there were no additional network elements in the communication path. Therefore, in the case of this measurement setup, some distortion aspects were eliminated. Figure 16 presents the e2e RTT results, where the server was connected directly to the SGi interface, while, on the client-side, the UE (5G modem) was connected to a 5G macrocell. Table 4 summarizes the latency results of these tests.

These measurements aimed to show what RTT values can be achieved with the currently available 5G modem on a commercial 5G network. With 40 Bytes packets, 9 ms RTT can be reached stably without any parameter tuning, such as TDD ratio tuning. Similarly to the M86 latency results, the median and average increase if the packet length rises, even in non-segmented packets (smaller than 1450 Bytes). Additionally, the latency distribution becomes significantly more spread based on the Q3 and max score.

Notice again, that the on-way-delay for these symmetrical UL/DL cases can be considered to be half of the RTT. This brings the average one-way-delay below 5 ms, in real-life measurements, even without network slicing applied. Hence, the presented measurement results and available 5G NSA network solutions are very promising and ready to support several V2X use-cases. Some of them are presented in Table 5 based on 3GPP V2X requirements [14], where the communication scenario, the degree of automation, and some key technical parameters (latency, data rate, range) are discussed.

The different categories of communication scenarios are as follows:

- Category 1—Vehicles Platooning enables the vehicles to dynamically form a group travelling together.
- Category 2—Advanced Driving enables semi-automated or fully-automated driving.
- Category 3—Extended Sensors enables the exchange of raw or processed data gathered through local sensors or live video data among vehicles, RSUs, devices of pedestrians, and V2X application servers.

In summary, a wide scope of V2X use-cases can be already achieved mostly for the lower degree of automation scenarios that are described in [14], which is the guiding 3GPP standard. These includes cooperative driving for vehicle platooning, reporting, and information sharing related to platooning, cooperative lane change, intersection safety, and various sorts of information sharing for automated driving and otherwise. Table 5 puts these scenarios into the perspective of the standard.

## 7. Conclusions

This paper presented a series of 5G-supported autonomous driving demonstrations that were accomplished in and around the ZalaZONE proving ground. The demonstrations used the Scenario-in-the-Loop method, where parts of the obstacles and decision-triggering information were arriving from the Digital Twin of the physical vehicle.

The demonstrations themselves were successful with 5G-based communication operating successfully on the live network. The self-driving car communicated with its Digital Twin and successfully executed the planned SCiL tests, e.g., braking in front of virtual and real pedestrians, tracking, and dodging virtual and real cars. The extension of the demonstration was carried out on the M86 national highway, where the vehicles run at various speeds and executed different tasks—and all related positioning and RSU data were recorded. To support future SciL tests, this sensor data can be re-played to put the Digital Twin into the virtual M86 environment.

In order to evaluate current 5G capabilities in real-life scenarios, we have carried out a series of 5G NSA option 3.X architecture measurements, addressing Round-Trip-Time and packet Inter-Arrival Time distributions. These helped to identify latency capabilities of current 5G NSA network capabilities. We found that various V2X scenarios—from platooning to intersection information exchange and many more—can already be supported according to standard requirements.

During the complex demonstration compiled with the wide-ranging cooperation of educational and industrial partners, it was one of the first times in the world (in May 2019) to see how the advantages of the low-latency 5G mobile network can be used to drive self-driving cars. Additionally, for the first time in Hungary, Magyar Telekom has created a real 5G test environment in ZalaZONE. Testing of the 5G NSA station built on the test track in collaboration with Ericsson has been ongoing since spring 2018. Another milestone of this work was the launch of the standard 5G service in May 2019 and demonstrations with partners.

All of this became the leading domestic news in connection with the completion of the first phase of the ZalaZONE project. As the task requires the integration of competencies across departments and faculties, the research team included researchers and developers from several departments and faculties, as well as consulting with leading researchers from around the world.

The demonstration’s significance is that it took place in real and virtual space at the same time. The vehicle can react to obstacles and traffic situations in both virtual and real space. With this solution, the development and testing of self-driving systems can be faster and more cost-effective. Testing in the real environment can only begin if the given functions and systems already work properly in the simulation environment.

There are various future directions of this research, both for the SciL topic, the 5G V2X evaluations, as well as the integration of these domains. The further steps regarding the SciL methodology include the creation, validation, and verification of further—more complex—traffic cases, as well as recording various scenarios for the Digital Twin to re-play. There are various enhancement possibilities arise with enriched environmental sensor communications—scenarios include communication with the traffic control systems [42], or further scenarios described in [34]. Regarding 5G V2X benchmarking, the most important further steps are the latency measurements of the upcoming 5G standalone architecture (with pure 5G core), where the end-to-end service slice can be dedicated for the V2X communication. Further, related research gaps and directions on ultra-reliable low latency communications are discussed in [10].

## Figures and Tables

**Table 1:** Requirements for messages of hard-real time and soft-real time data transfer within SciL.

Requirements	Hard-Real-Time	Soft-Real-Time
**Typical response time**	milliseconds range	seconds range
**Behaviour at peak load**	Well-defined, prioritised	Best effort, problems can occur at peak loads
**Timing control**	Deterministic, quasi-synchronous	Depending on the environment, non-deterministic response time
**Safety and** **error detection**	Autonomous error detection	Operator intervention
**Typical size of data**	Small (tens of Bytes)	Big (kBytes, or greater streams)

**Table 2:** Data types and used sensor devices, where GNSS is the Global Navigation Satellite System, and IMU is the Inertial Measurement Unit.

Type of Data	Sensor Device
GNSS coordinates	GNSS
Direction	GNSS/IMU
Velocity	GNSS/IMU/ECU
Acceleration	GNSS/IMU/ECU
Dynamic objects	Camera/Lidar/Radar/V2X
Lane position	Camera/GNSS
Lane geometry	Camera
Neighbour lanes	Camera

**Table 3:** Aggregated (median) results of comparative measurements  [23].

	4G—Only	5G—1 UE	5G—2 UE
**Downlink Peak Rate**	420 Mbps	885 Mbps	1465 Mbps
**Uplink Peak Rate**	87 Mbps	92 Mbps	91 Mbps
**Latency (one-way)**	12 ms	3.71 ms	4.96 ms
**Packet Error Rate**	0.2%	0.39%	0.67%

**Table 4:** Public network statistics of the e2e 5G RTT.

Packet Length	Minimum	Average	Maximum	Standard Deviation
**40 Byte**	6.958 ms	9.247 ms	25.135 ms	1.486 ms
**100 Byte**	10.002 ms	12.739 ms	25.796 ms	1.463 ms
**200 Byte**	10.511 ms	12.837 ms	32.174 ms	1.489 ms
**500 Byte**	13.095 ms	15.380 ms	34.815 ms	1.560 ms
**1000 Byte**	13.223 ms	15.470 ms	136.886 ms	3.280 ms
**2000 Byte**	14.944 ms	18.580 ms	129.536 ms	3.883 ms

**Table 5:** Achievable V2X scenarios with current solutions—based on 3GPP TS 22.186  [14].

Communication Scenario	Degree	Max E2E Latency [ms]	Data Rate [Mbps]	Min Range [m]
Cooperative driving for vehicle platooning [1]	*Lowest degree of* *automation*	25	–	–
	*Low degree of* *automation*	20	–	350
	*High degree of* *automation*	20	65	180
Reporting for platooning [1]	*N/A*	500	–	–
Information sharing for platooning [1]	*Lower degree of* *automation*	20	–	350
	*Higher degree of* *automation*	20	50	180
Inf. sharing for automated driving supporting V2X [2]	*Lower degree of* *automation*	100	–	700
	*Higher degree of* *automation*	100	10	360
Inf. sharing for automated driving supporting V2X and RSU [2]	*Lower degree of* *automation*	100	–	700
	*Higher degree of* *automation*	100	53	360
Intersection safety information [2]	*–*	–	UL: 0. 25 DL: 50 (NOTE 1)	–
Cooperative lane change [2]	*Lower degree of* *automation*	25	–	–
Sensor information sharing [3]	*Lower degree of* *automation*	100	–	1000
Video sharing [3]	*Lower degree of* *automation*	90	10	100

NOTE 1: This value is referring to a maximum number of 200 UEs. The value of 50 Mbps DL is applicable to broadcast or is the maximum aggregated bitrate of the all UEs for unicast. Requirement categories: [1]—Vehicles Platooning, [2]—Advanced Driving, [3]—Extended Sensors.

## References

[1] Feddes G., Kuipers J. (2018). Software Driving License.

[2] ZalaZONE Where Innovation Leads. https://zalazone.hu/en/.

[3] Szalay Z., Hamar Z., Nyerges A. (2019). Novel design concept for an automotive proving ground supporting multilevel CAV development. Int. J. Veh. Des..

[4] Horváth M., Lu Q., Tettamanti T., Török A., Szalay Z. (2020). Vehicle-In-The-Loop (VIL) and Scenario-In-The-Loop (SCIL) Automotive Simulation Concepts from the Perspectives of Traffic Simulation and Traffic Control. Transp. Telecommun. J..

[5] Tihanyi V., Szalay Z. Autonomous vehicle platform for demonstration purposes. Proceedings of the Advanced Manufacturing and Repairing Technologies in Vehicle Industry.

[6] Kozma D., Soos G., Ficzere D., Varga P. Communication Challenges and Solutions between Heterogeneous Industrial IoT Systems. Proceedings of the 2019 15th International Conference on Network and Service Management (CNSM).

[7] Németh H., Háry A., Szalay Z., Tihanyi V., Tóth B., Proff H. (2019). Proving Ground Test Scenarios in Mixed Virtual and Real Environment for Highly Automated Driving. Mobilität in Zeiten der Veränderung: Technische und Betriebswirtschaftliche Aspekte.

[8] Heineke K., Ménard A., Södergren F., Wrulich M. (2019). Development in the Mobility Technology Ecosystem—How Can 5G Help? McKinsey and Company. https://www.mckinsey.com/industries/automotive-and-assembly/our-insights/development-in-the-mobility-technology-ecosystem-how-can-5g-help.

[9] Ge X. (2019). Ultra-Reliable Low-Latency Communications in Autonomous Vehicular Networks. IEEE Trans. Veh. Technol..

[10] Varga P., Peto J., Frankó A., Balla D., Haja D., Janky F., Soos G., Ficzere D., Maliosz M., Toka L. (2020). 5G support for Industrial IoT Applications—Challenges, Solutions, and Research gaps. Sensors.

[11] Soós G., Ficzere D., Varga P., Szalay Z. Practical 5G KPI Measurement Results on a Non-Standalone Architecture. Proceedings of the NOMS 2020—2020 IEEE/IFIP Network Operations and Management Symposium.

[12] 3GPP (2019). Service Requirements for the 5G System.

[13] 3GPP (2019). Service Requirements for Cyber-Physical Control Applications in Vertical Domains.

[14] 3GPP (2019). Service Requirements for Enhanced V2X Scenarios.

[15] 3GPP (2019). Mobile Communication System for Railways.

[16] 3GPP (2019). System Architecture for the 5G System (5GS); Stage 2.

[17] Afaq M., Iqbal J., Ahmed T., Ul Islam I., Khan M., Khan M.S. (2020). Towards 5G network slicing for vehicular ad-hoc networks: An end-to-end approach. Comput. Commun..

[18] ONF (2020). Converged Multi-Access and Core (COMAC).

[19] Yu R., Ding J., Huang X., Zhou M., Gjessing S., Zhang Y. (2016). Optimal Resource Sharing in 5G-Enabled Vehicular Networks: A Matrix Game Approach. IEEE Trans. Veh. Technol..

[20] Balasubramanian V., Otoum S., Aloqaily M., Al Ridhawi I., Jararweh Y. (2020). Low-latency vehicular edge: A vehicular infrastructure model for 5G. Simul. Model. Pract. Theory.

[21] Li G., Lai C. Platoon Handover Authentication in 5G-V2X: IEEE CNS 20 Poster. Proceedings of the 2020 IEEE Conference on Communications and Network Security (CNS).

[22] Ullah H., Gopalakrishnan Nair N., Moore A., Nugent C., Muschamp P., Cuevas M. (2019). 5G Communication: An Overview of Vehicle-to-Everything, Drones, and Healthcare Use-Cases. IEEE Access.

[23] Soós G., Ficzere D., Varga P. (2020). Towards Traffic Identification and Modeling for 5G Application Use-Cases. Electronics.

[24] Isto P., Heikkilä T., Mämmelä A., Uitto M., Seppälä T., Ahola J.M. (2020). 5G Based Machine Remote Operation Development Utilizing Digital Twin. Open Eng..

[25] Cao H., Gangakhedkar S., Ali A.R., Gharba M., Eichinger J. A 5G V2X testbed for cooperative automated driving. Proceedings of the 2016 IEEE Vehicular Networking Conference (VNC).

[26] Maskulainen I., Luoto P., Pirinen P., Bennis M., Horneman K., Latva-aho M. Performance evaluation of adaptive beamforming in 5G-V2X networks. Proceedings of the 2017 European Conference on Networks and Communications (EuCNC).

[27] Barmpounakis S., Tsiatsios G., Papadakis M., Mitsianis E., Koursioumpas N., Alonistioti N. (2020). Collision avoidance in 5G using MEC and NFV: The vulnerable road user safety use case. Comput. Netw..

[28] Wang P., Di B., Zhang H., Bian K., Song L. (2019). Platoon Cooperation in Cellular V2X Networks for 5G and Beyond. IEEE Trans. Wirel. Commun..

[29] Kiela K., Barzdenas V., Jurgo M., Macaitis V., Rafanavicius J., Vasjanov A., Kladovscikov L., Navickas R. (2020). Review of V2X–IoT Standards and Frameworks for ITS Applications. Appl. Sci..

[30] Lee W., Na T., Kim J. How to Create a Network Slice? —A 5G Core Network Perspective. Proceedings of the 2019 21st International Conference on Advanced Communication Technology (ICACT).

[31] Srinivasa R., Naidu N.K.S., Maheshwari S., Bharathi C., Hemanth Kumar A.R. Minimizing Latency for 5G Multimedia and V2X Applications using Mobile Edge Computing. Proceedings of the 2019 2nd International Conference on Intelligent Communication and Computational Techniques (ICCT).

[32] Husain S.S., Kunz A., Prasad A., Pateromichelakis E., Samdanis K. (2019). Ultra-High Reliable 5G V2X Communications. IEEE Commun. Stand. Mag..

[33] Hawkins A.J. Waymo Gets the Green Light to Test Fully Driverless Cars in California. https://www.theverge.com/2018/10/30/18044670/waymo-fully-driverless-car-permit-california-dmv.

[34] Wang J., Shao Y., Ge Y., Yu R. (2020). A Survey of Vehicle to Everything (V2X) Testing. Sensors.

[35] Park C., Chung S., Lee H. (2020). Vehicle-in-the-Loop in Global Coordinates for Advanced Driver Assistance System. Appl. Sci..

[36] Horváth M., Tettamanti T., Varga B., Szalay Z. The Scenario-in-the-Loop (SciL) automotive simulation concept and its realisation principles for traffic control. Proceedings of the 8th Symposium of the European Association for Research in Transportation.

[37] Eichberger A., Markovic G., Magosi Z., Rogic B., Lex C., Samiee S. (2017). A Car2X sensor model for virtual development of automated driving. Int. J. Adv. Robot. Syst..

[38] Pariota L., Bifulco G.N., Markkula G., Romano R. Validation of driving behaviour as a step towards the investigation of Connected and Automated Vehicles by means of driving simulators. Proceedings of the 2017 5th IEEE International Conference on Models and Technologies for Intelligent Transportation Systems (MT-ITS).

[39] Szalay Z., Szalai M., Tóth B., Tettamanti T., Tihanyi V. Proof of concept for Scenario-in-the-Loop (SciL) testing for autonomous vehicle technology. Proceedings of the 2019 IEEE International Conference on Connected Vehicles and Expo (ICCVE).

[40] ASAM ASAM OpenSCENARIO. https://www.asam.net/standards/detail/openscenario/.

[41] GSMA (2020). 5G Implementation Guidelines: NSA Option 3.

[42] Alekszejenko L., Dobrowiecki T. (2020). Adapting IT Algorithms and Protocols to an Intelligent Urban Traffic Control. Inforcommun. J..

